# Prenatal pet keeping and caregiver-reported attention deficit hyperactivity disorder through preadolescence in a United States birth cohort

**DOI:** 10.1186/s12887-019-1719-9

**Published:** 2019-10-29

**Authors:** Andrea E. Cassidy-Bushrow, Alexandra R. Sitarik, Tisa M. Johnson-Hooper, Jannel M. Phillips, Kyra Jones, Christine Cole Johnson, Jennifer K. Straughen

**Affiliations:** 1 Department of Public Health Sciences, Henry Ford Hospital, 1 Ford Place, 5C, Detroit, MI 48202 USA; 2 Center for Urban Responses to Environmental Stressors, Wayne State University, Detroit, MI 48202 USA; 3 Department of Pediatrics, Henry Ford Hospital, 2799 West Grand Blvd, Detroit, MI 48202 USA; 4 Center for Autism and Developmental Disabilities, Henry Ford Hospital, 2799 West Grand Blvd, Detroit, MI 48202 USA; 5 Department of Psychiatry and Behavioral Health Services, Division of Neuropsychology, Henry Ford Hospital, 2799 West Grand Blvd, Detroit, MI 48202 USA

**Keywords:** Pet keeping, Attention deficit hyperactivity disorder, Prenatal, Birth cohort

## Abstract

**Background:**

While the keeping of pets has been shown to protect against childhood allergic disease and obesity, less is known regarding potential associations of prenatal pet keeping and attention deficit hyperactivity disorder (ADHD). We sought to examine the associations between prenatal dog or cat keeping with caregiver-reported ADHD in preadolescents in the Wayne County Health, Environment, Allergy and Asthma Longitudinal Study (WHEALS) birth cohort (*N* = 1258).

**Methods:**

At an interview with the caregiver at child age 10–12 years, caregivers reported if the WHEALS child had ever been diagnosed with ADHD. Similarly, during an interview with the mother prenatally, pet keeping (defined as dog or cat kept inside ≥1 h/day) was ascertained. Logistic regression models were fit to examine the association of prenatal pet keeping (dog keeping and cat keeping, separately) with ADHD.

**Results:**

A subset of 627 children were included in the analyses: 93 who had ADHD and 534 with neurotypical development. After accounting for confounders and loss to follow-up, maternal prenatal dog exposure was associated with 2.23 times (95% CI: 1.15, 4.31; *p* = 0.017) greater odds of ADHD among boys. Prenatal dog keeping was not statistically significantly associated with ADHD in girls (odds ratio = 0.27, 95% CI: 0.06, 1.12; *p* = 0.070). Prenatal cat keeping was not associated with ADHD.

**Conclusions:**

In boys, but not girls, maternal prenatal dog keeping was positively associated with ADHD. Further study to confirm these findings and to identify potential mechanisms of this association (e.g., modification of the gut microbiome, exposure to environmental toxicants or pet-related medications) is needed.

## Background

The keeping of pets has been shown to have a variety of both positive and negative health effects [1–8]. Children exposed to pets during early life are less likely to develop allergic diseases as well as obesity [9–13]. However, little is known regarding the association between pet keeping and neurodevelopmental disorders, including attention deficit hyperactivity disorder (ADHD), with limited published studies showing positive and null associations between pet keeping and neurodevelopmental disorders or measures of related symptoms [14–16].

Pets may influence neurodevelopment via several pathways. Pets are associated with changes in the gut microbiome of members of their household [17, 18]. The gut microbiome may influence risk of ADHD via the gut-brain axis [19–21]. Additionally, pets may introduce environmental toxicants (e.g., pesticides) into the home [22], and pesticide exposure may be a risk factor for ADHD [23]. Finally, attachment to a pet may impact emotional development [24]; emotional dysregulation is a feature of ADHD [25].

ADHD is a complex disorder hallmarked by hyperactivity, inattention and impulsivity at a developmentally inappropriate level [26]. In the United States, ~ 9.0% of children are affected by ADHD [27]. While there is a large genetic component to ADHD (estimated heritability > 70%) [28], environmental factors also impact the risk of ADHD [29]. Given that ADHD results in a sizeable economic burden [30] and negative impact on quality of life [31], there is a need for further study of potential environmental risk factors, particularly those in early life [32].

The goal of this study was to examine if prenatal pet keeping was associated with ADHD in preadolescence (ages ~ 10–12 years). To achieve this goal, we leveraged data from the racially and socioeconomically diverse Wayne County Health, Environment, Allergy and Asthma Longitudinal Study (WHEALS) birth cohort (Detroit, Michigan) [11, 33, 34].

## Methods

### Study population

WHEALS recruited pregnant women with due dates from September 2003 through December 2007, and who were seeing a practitioner at 1 of 5 clinics in the Henry Ford Health System to establish a birth cohort [11, 34]. All women were in their second trimester or later, were aged 21–49 years, and were living in a predefined geographic area in western Wayne County that included the western portion of the city of Detroit as well as the suburban areas immediately surrounding the city. Mothers were interviewed in the clinic prenatally and postpartum interviewer-administered questionnaires were completed at child age 1, 6, 12, and 24 months. Children and their parent/guardian were invited to return for a clinic visit at child age 2 years and again at child age 10–12 years for assessment of child health. All participants provided written, informed consent and study protocols were approved by the Henry Ford Health System Institutional Review Board; at the age 10–12 year visit, children provided written, informed assent.

### Definition of ADHD, other neurodevelopmental disorders, and Neurotypical development

At the age 10–12 year visit, the caregiver (95% the mother) reported if the child had ever been diagnosed with ADHD. The caregiver also reported if the child had ever been diagnosed with autism spectrum disorder (ASD), Asperger’s syndrome, or sensory processing disorder (SPD); Asperger’s syndrome was asked separately since children may have been diagnosed prior to the Diagnostic and Statistical Manual of Mental Disorders, Fifth Edition (DSM-5). A report of “suspect” diagnosis was also considered positive. Children were considered neurotypical (NT) if they did not have a caregiver-reported ASD, Asperger’s, ADHD, or SPD diagnosis. Children without an ADHD diagnosis but who had a caregiver-reported ASD, Asperger’s or SPD diagnosis were excluded from the analysis, given the small sample size with these conditions.

A subset of 325 WHEALS children had their medical record abstracted for additional health information, including ADHD diagnosis. Caregiver report of ADHD diagnosis was validated within this subset using the kappa (κ) statistic to evaluate agreement. Landis and Koch [35] criteria were used to evaluate strength of agreement. There was near perfect agreement between caregiver-reported ADHD and the medical record (κ = 0.84, 95% CI 0.78, 0.91). Given that only a subset of children had medical record review and the high level of agreement between caregiver report and the medical record, we utilized caregiver-reported ADHD in statistical analysis.

### Prenatal pet keeping

During the prenatal maternal interview, the mother was asked about presence of pets in the home, the number of hours they spent indoors, and the type of pet (e.g., dog or cat). Some studies have attributed health benefits primarily to dog ownership [2, 36, 37]; thus, we defined prenatal dog keeping and prenatal cat keeping seperately, as report of having a dog or cat, respectively, indoors at least 1 h per day.

### Covariates

The mother self-reported race, date of birth, marital status, household income, education, parity, smoking history and exposure to environmental tobacco smoke at the prenatal interview. Maternal prenatal care records were abstracted to obtain antibiotic and antifungal use [38]. Height and weight were also abstracted and body mass index (BMI) at first prenatal care visit (mean gestational age at measure 9.1 ± 4.9 weeks; 83% taken during the first trimester) was calculated. Although first measured BMI in pregnancy and self-reported prepregnancy BMI have been shown to be highly correlated in other studies [39], the first measured BMI during pregnancy represents both prepregnancy body size and early pregnancy-related weight gain. Delivery records for WHEALS women were abstracted to obtain delivery mode, birth weight, and gestational age at delivery. Low birth weight was defined as a birthweight < 2500 g and preterm delivery as gestational age at delivery < 37 weeks.

### Statistical analysis

Differences in basic characteristics by ADHD vs. NT development were first compared using standard parametric statistical methods, including chi-square tests and analysis of variance. Prenatal pet exposure (dogs and cats, separately) was then associated with ADHD using logistic regression to obtain odds ratios (OR) and 95% CIs. Given the growing and consistent evidence that male fetuses are more vulnerable than female fetuses to exposures during gestation [40], sex-specific effects of pets on ADHD was a priori hypothesized and formally tested using interaction terms in logistic regression models. When the interaction term was statistically significant, sex-specific models were fit to aid in interpretation.

Not all WHEALS children/caregiver pairs completed a 10–12 year questionnaire (a comparison of those with and without 10–12 year questionnaire data is presented in Table 1). Inverse probability weights (IPW) [41–43] were used to account for loss to follow-up by age 10–12 years, defined by completion of the 10–12-year questionnaire. Weights were calculated as the inverse of the “treatment” received. In other words, if p = probability of follow-up, then w = 1/p for children with follow-up, and w = 1/(1-p) for children without follow-up. Briefly, IPWs up weight the importance of children who were followed through age 10–12 years but were not very likely to have been followed based on baseline characteristics (e.g., children of mothers who smoked during pregnancy). Similarly, it down weights the importance of children who were followed through age 10–12 years but were very likely to have been followed (e.g., children of mothers with a high level of education). The following factors were included in the calculation of the IPWs: maternal race, insurance, household income, maternal education, firstborn child, maternal smoking during pregnancy, prenatal environmental tobacco smoke exposure, urban residence, marital status, mode of delivery, prenatal alcohol use, child sex, prenatal dogs, prenatal cats, maternal history of allergies and asthma, maternal age at delivery, gestational age at delivery, birthweight, and completion of the 2-year clinic visit. Balance in these covariates was assessed using standardized differences before and after weighting, with imbalance defined as absolute value > 0.20. In addition to weighting the models to account for loss to follow-up, models were additionally adjusted for potentially confounding covariates. Confounders were selected based on having a significant association with either prenatal pet keeping or ADHD. These adjustment covariates included maternal race, household income, maternal smoking during pregnancy, prenatal antifungal use, child sex, maternal BMI first measured in pregnancy, gestational age at delivery, and birthweight.

**Table 1:** Differences in maternal and child characteristics in Wayne County Health, Environment, Allergy and Asthma Longitudinal Study participants with and without the 10–12 year questionnaire data, before and after inverse probability weighting (IPW)

Covariate		Completed the 10–12-Year Questionnaire		Before IPW		After IPW	
		No *N* = 613	Yes *N* = 645	P^a^	D^b^	P^a^	D^b^
Maternal Characteristic		Mean ± SD or *N* (%)					
Age at delivery (years)		28.9 ± 5.1	30.2 ± 5.3	**< 0.001**	0.25	0.642	0.02
Race	*White*	133 (21.7%)	157 (24.3%)	0.570	0.10	0.962	0.03
	*Black*	387 (63.1%)	391 (60.6%)				
	*Hispanic*	40 (6.5%)	38 (5.9%)				
	*Arabic*	31 (5.1%)	28 (4.3%)				
	*Mixed/Other*	22 (3.6%)	31 (4.8%)				
Marital status	*Unmarried*	273 (44.5%)	212 (32.9%)	**< 0.001**	0.24	0.335	0.04
	*Married*	340 (55.5%)	433 (67.1%)				
Insurance coverage	*HAP*	169 (27.6%)	333 (51.6%)	**< 0.001**	0.71	0.815	0.04
	*Other insurance*	198 (32.3%)	233 (36.1%)				
	*No insurance*	9 (1.5%)	6 (0.9%)				
	*Refused/do not know/missing*	237 (38.7%)	73 (11.3%)				
Household income	*<$20,000*	112 (18.3%)	70 (10.9%)	**< 0.001**	0.32	0.938	0.05
	*$20,000- < $40,000*	150 (24.5%)	145 (22.5%)				
	*$40,000- < $80,000*	173 (28.2%)	174 (27.0%)				
	*$80,000- < $100,000*	49 (8.0%)	86 (13.3%)				
	*≥$100,000*	52 (8.5%)	96 (14.9%)				
	*Refused to answer*	77 (12.6%)	74 (11.5%)				
Education	*<HS diploma*	50 (8.2%)	24 (3.7%)	**< 0.001**	0.41	0.967	0.02
	*HS diploma*	131 (21.4%)	97 (15.0%)				
	*Some college*	313 (51.1%)	292 (45.3%)				
	*≥Bachelor’s degree*	119 (19.4%)	232 (36.0%)				
Mother smoked during pregnancy	*No*	517 (84.3%)	591 (91.6%)	**< 0.001**	− 0.23	0.420	− 0.03
	*Yes*	96 (15.7%)	54 (8.4%)				
ETS during pregnancy	*No*	421 (68.7%)	490 (76%)	**0.004**	−0.16	0.666	−0.02
	*Yes*	192 (31.3%)	155 (24%)				
Prenatal alcohol use	*No*	590 (96.6%)	616 (95.7%)	0.405	0.05	0.330	−0.04
	*Yes*	21 (3.4%)	28 (4.3%)				
Location of residence	*Suburban*	250 (40.8%)	305 (47.3%)	**0.020**	−0.13	0.224	−0.05
	*Urban*	363 (59.2%)	340 (52.7%)				
Doctor-diagnosed hay fever or allergic rhinitis	*No*	516 (85.0%)	538 (84.9%)	0.941	0.001	0.613	0.02
	*Yes*	91 (15.0%)	96 (15.1%)				
Doctor-diagnosed asthma	*No*	492 (80.3%)	513 (79.7%)	0.790	0.01	0.584	0.02
	*Yes*	121 (19.7%)	131 (20.3%)				
Prenatal indoor dog(s)	*No*	476 (77.7%)	479 (74.3%)	0.160	0.08	0.797	−0.01
	*Yes*	137 (22.3%)	166 (25.7%)				
Prenatal indoor cat(s)	*No*	520 (84.8%)	535 (82.9%)	0.364	0.05	0.538	0.02
	*Yes*	93 (15.2%)	110 (17.1%)				
Clinical Characteristics							
Mode of delivery	*Vaginal*	385 (63.4%)	399 (62.1%)	0.616	0.03	0.513	−0.03
	*C-section*	222 (36.6%)	244 (37.9%)				
Firstborn	*No*	401 (65.4%)	397 (61.6%)	0.155	0.08	0.986	−0.001
	*Yes*	212 (34.6%)	248 (38.4%)				
Child sex	*Male*	308 (50.3%)	314 (48.7%)	0.560	0.03	0.557	0.02
	*Female*	304 (49.7%)	331 (51.3%)				
Gestational age at delivery (weeks)^c^		38.7 ± 1.8 (*N* = 595)	38.8 ± 1.7 (*N* = 637)	0.307	0.05	0.902	0.004
Birthweight (grams)^c^		3258 ± 548 (*N* = 567)	3346 ± 594 (*N* = 615)	**0.008**	0.15	0.880	−0.006
Completed 2-year clinic visit	*No*	425 (69.3%)	137 (21.2%)	**< 0.001**	1.10	0.596	0.02
	*Yes*	188 (30.7%)	508 (78.8%)				

*ETS* environmental tobacco smoke, *HAP* Health Alliance Plan, *HS* high school, *SD* standard deviation

^a^Calculated using chi-square test for categorical covariates and analysis of variance for continuous covariates. Bold values indicate statistically significant *P*<0.05

^b^Standardized difference (difference in means or proportions divided by standard error); imbalance defined as absolute value> 0.20

^c^Continuous covariates with missing data, sample size is presented

If complete case analysis were used in the adjusted models, approximately 25% of the data would be excluded. To better handle this missingness, multiple imputation was used; all adjustment covariates as well as exposures (prenatal dogs/cats) and outcome (ADHD) were used to calculate 25 total imputed datasets. Each imputed dataset was modeled using logistic regression (with IPWs normalized to sum to the actual sample size as well as confounders included); final effect estimates were obtained by pooling results. All analyses were performed using SAS version 9.4 (SAS Institute Inc., Cary, NC) and R version 3.4.1.

## Results

Of the original 1258 WHEALS children, 645 caregivers completed a 10–12 year questionnaire. Of these, 2 did not complete the neurodevelopmental disorder section of the questionnaire. Children with ASD/Asperger’s (*n* = 6), SPD (*n* = 7), or both (*n* = 3) without an ADHD diagnosis were excluded. Therefore, our final analytic sample consisted of 627 children: 93 with an ADHD diagnosis and 534 NT children. Differences in those who did and did not complete the 10–12 year questionnaire data are presented in Table 1. Briefly, mothers who completed the questionnaire were older and were more likely to be married, have higher household incomes, have higher levels of education, and were more likely to live in the suburbs (all *p* < 0.05). Additionally, they were less likely to smoke prenatally and be exposed to environmental tobacco smoke prenatally; the children of these mothers were also heavier at birth and were more likely to complete the 2-year clinic visit (all *p* < 0.05). However, though the standardized differences (D) of these effects were often large, inverse probability weighting adequately removed these imbalances (Table 1; absolute value of all D < 0.05 after IPW).

Table 2 presents descriptive characteristics by ADHD vs NT. As expected, boys were more likely to have ADHD than girls (*p* < 0.001). Children with ADHD were more likely to have mothers that used antifungals prenatally (*p* = 0.004) and who had a higher BMI recorded at the first prenatal visit (*p* = 0.002). Additionally, children with ADHD had an earlier mean gestational age at delivery (38.3 ± 2.2 weeks compared to 38.8 ± 1.6 weeks; *p* = 0.007) and were more likely to have been born preterm than NT children (*p* = 0.018).

**Table 2:** Descriptive characteristics of Wayne County Health, Environment, Allergy and Asthma Longitudinal Study children with caregiver-reported attention deficit hyperactive disorder (ADHD) or neurotypical (NT) development

Covariate	NT (*N* = 534)	ADHD (*N* = 93)	*P* ^*a*^
Maternal Characteristic	Mean ± SD or *N* (%)		
Age at delivery (years)	30.1 ± 5.2	30.0 ± 5.3	0.814
Race			0.980
*White*	127 (23.8%)	23 (24.7%)	
*Black*	325 (60.9%)	56 (60.2%)	
*Mixed/Other*	82 (15.4%)	14 (15.1%)	
Marital status			0.634
*Unmarried*	176 (33.0%)	33 (35.5%)	
*Married*	358 (67.0%)	60 (64.5%)	
Household income			0.208
*< $20,000*	53 (9.9%)	14 (15.1%)	
*$20,000- < $40,000*	118 (22.1%)	25 (26.9%)	
*$40,000- < $80,000*	142 (26.6%)	27 (29.0%)	
*$80,000- < $100,000*	75 (14.0%)	10 (10.8%)	
*≥ $100,000*	83 (15.5%)	7 (7.5%)	
*Refused to answer*	63 (11.8%)	10 (10.8%)	
Education			0.097
*< High school diploma*	20 (3.7%)	2 (2.2%)	
*High school diploma*	81 (15.2%)	14 (15.1%)	
*Some college*	235 (44.0%)	53 (57.0%)	
*≥ Bachelor’s degree*	198 (37.1%)	24 (25.8%)	
Smoked during pregnancy			0.600
*No*	491 (91.9%)	84 (90.3%)	
*Yes*	43 (8.1%)	9 (9.7%)	
Prenatal ETS exposure			0.303
*No*	411 (77.0%)	67 (72.0%)	
*Yes*	123 (23.0%)	26 (28.0%)	
Prenatal antibiotic use			0.774
*No*	192 (45.2%)	38 (46.9%)	
*Yes*	233 (54.8%)	43 (53.1%)	
Prenatal antifungal use			**0.004**
*No*	356 (83.8%)	57 (70.4%)	
*Yes*	69 (16.2%)	24 (29.6%)	
BMI from first prenatal visit (kg/m^2^)^b^	30.3 ± 7.7 (*N* = 495)	33.1 ± 9.6 (*N* = 87)	**0.002**
Prenatal indoor dog(s)			0.308
*No*	400 (74.9%)	65 (69.9%)	
*Yes*	134 (25.1%)	28 (30.1%)	
Prenatal indoor cat(s)			0.863
*No*	444 (83.1%)	78 (83.9%)	
*Yes*	90 (16.9%)	15 (16.1%)	
Child Characteristic			
Mode of delivery			0.513
*Vaginal*	332 (62.3%)	54 (58.7%)	
*C-section*	201 (37.7%)	38 (41.3%)	
Firstborn			0.343
*No*	332 (62.2%)	53 (57.0%)	
*Yes*	202 (37.8%)	40 (43.0%)	
Gestational age at delivery (weeks)^b^	38.8 ± 1.6 (*N* = 529)	38.3 ± 2.2 (*N* = 91)	**0.007**
Preterm birth			**0.018**
*No*	492 (93.0%)	78 (85.7%)	
*Yes*	37 (7.0%)	13 (14.3%)	
Birthweight (g)^b^	3352 ± 573 (*N* = 514)	3300 ± 680 (*N* = 84)	0.453
Low birth weight			0.229
*No*	483 (94.0%)	76 (90.5%)	
*Yes*	31 (6.0%)	8 (9.5%)	
Child sex			**< 0.001**
*Male*	237 (44.4%)	69 (74.2%)	
*Female*	297 (55.6%)	24 (25.8%)	
Child age at 10–12-year visit (years)	10.3 ± 0.9	10.3 ± 1.0	0.646

*BMI* body mass index, *ETS* environmental tobacco smoke, *SD* standard deviation

^a^Calculated using chi-square test for categorical covariates and analysis of variance for continuous covariates. Bold values indicate statistically significant *P*<0.05

^b^Continuous covariates with missing data, sample size is presented

In unadjusted and unweighted analyses, children with ADHD were more likely to have had a mother exposed to dogs prenatally (30.1%) compared to children with NT development (25.1%), although this was not statistically significant (*p* = 0.308). There was no statistically significant difference in prenatal exposure to cats in children with ADHD compared to NT children (16.1% vs. 16.9%, respectively; *p* = 0.863).

After adjustment for loss to follow-up using IPW, the association between prenatal exposure to dogs and ADHD remained nonsignificant (*p* = 0.124), though the direction of association was the same (OR = 1.47, 95% CI: 0.90, 2.41; Table 3, Model 1). Results were similar after additional adjustment for potential confounding factors (Table 3, Model 2). Further, there was no statistically significant association between prenatal cat exposure and ADHD (Table 3; all *p* > 0.543).

**Table 3:** Association between prenatal indoor pet keeping and caregiver-reported attention deficit hyperactivity disorder

	Model 1^a^	Model 2^b^
	OR (95% CI)	OR (95% CI)
	*P*	*P*
Prenatal indoor dog(s)	1.47 (0.90, 2.41)	1.35 (0.77, 2.36)
	0.124	0.300
Prenatal indoor cat(s)	1.13 (0.64, 2.01)	1.23 (0.63, 2.39)
	0.666	0.543

*OR* odds ratio

^a^Model 1: inverse probability weights (IPW)

^b^Model 2: IPW + confounders, pooled estimate from multiple imputation model

Confounders: maternal race, household income, maternal smoking during pregnancy, prenatal antifungal use, child sex, maternal body mass index first measured in pregnancy, gestational age at birth, and birthweight

**Fig. 1:**
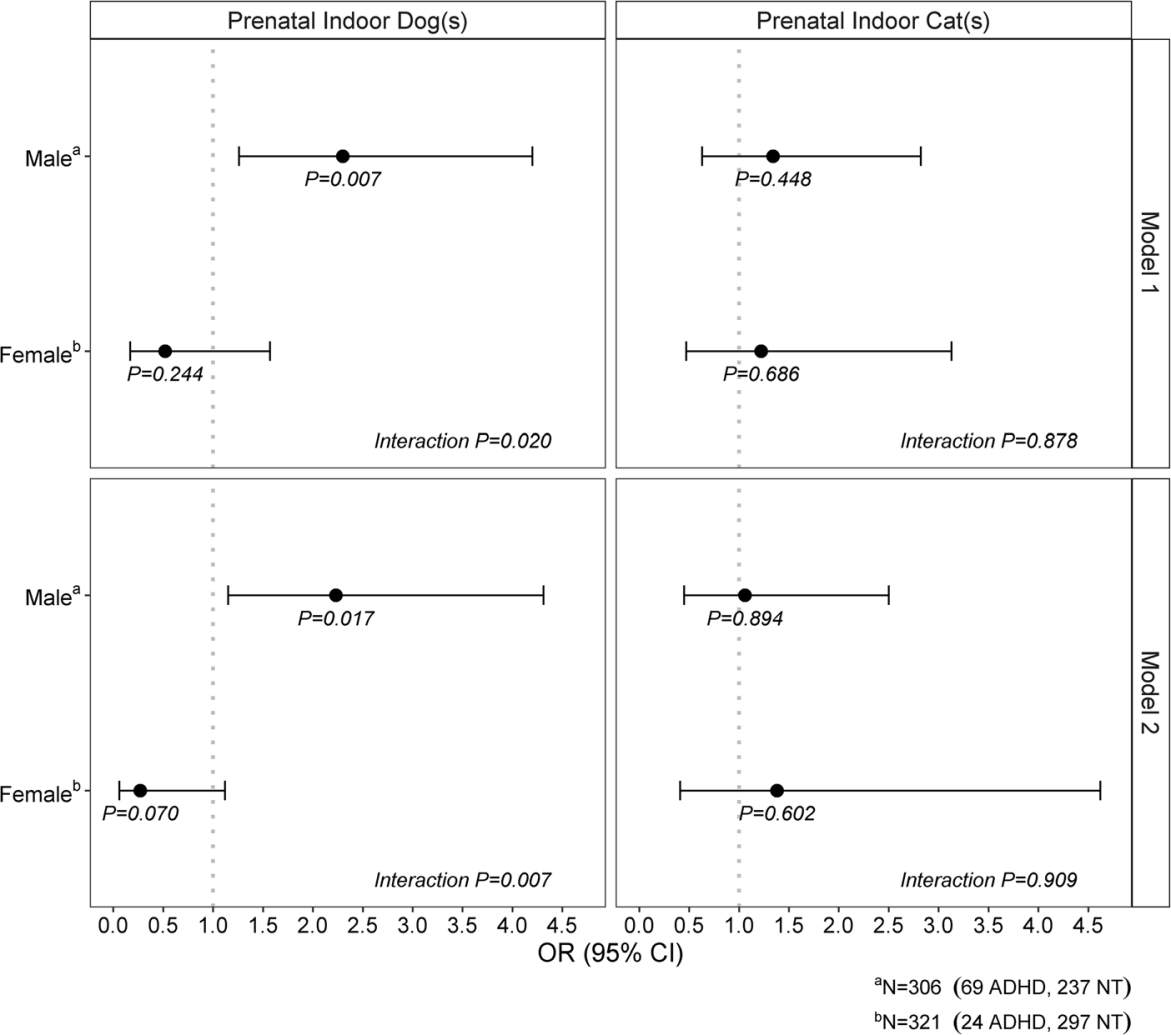
Sex-specific association between prenatal indoor pets and attention deficit hyperactivity disorder. Interaction *P*-value is for the child sex by pet keeping interaction term; sex-specific *P*-value is from models stratified by sex. Model 1 accounts for inverse probability weights (IPW); Model 2 accounts for IPW and confounders (pooled estimate from multiple imputation model). Confounders: maternal race, household income, maternal smoking during pregnancy, prenatal antifungal use, maternal body mass index first measured in pregnancy, gestational age at birth, and birthweight. Abbreviations: OR, odds ratio

When examining potential sex-specific effects of prenatal exposure to dogs on ADHD (Fig. 1), a significant interaction was found (Model 2; *p* = 0.007). Specifically, maternal prenatal exposure to dogs was statistically significantly associated with increased odds of ADHD among males (OR = 2.23, 95% CI: 1.15, 4.31, *p* = 0.017), but not females (OR = 0.27, 95% CI: 0.06, 1.12, *p* = 0.070). Conversely, the effect of cats on ADHD was not significantly modified by sex (Model 2; interaction *p* = 0.909; Fig. 1).

## Discussion

In the current study, prenatal exposure to dogs, but not cats, was associated with development of ADHD in male offspring. Nearly 3 times as many males as females are diagnosed with ADHD [44, 45]. Male fetuses appear to be more susceptible to negative effects of prenatal environmental exposures [40] and environmental exposures in early life not only influence the gut microbiome but also have sex-specific effects on the immune system [46]. Our findings that the association between prenatal dog keeping and ADHD is sex-specific suggest that future studies examining pets and ADHD should a priori consider evaluation of potential sex-specific effects.

Little is known about the keeping of pets during the prenatal period on risk of ADHD, although studies looking at childhood pet exposure with ADHD symptoms and/or diagnosis have found similar results. In the GINIPlus and LISAPlus German birth cohorts, ever owning a pet between birth and age 10 years was associated with higher scores for emotional symptoms and hyperactivity/impulsivity on the strengths and difficulties questionnaire at child age 10 years [14]. Similarly, in the 2003 California Health Interview Survey, currently allowing either a dog or cat into the house were positively associated with ADHD; however, this was slightly attenuated and no longer statistically significant after doubly robust adjustment for confounders (models adjusted for propensity score variables while also weighing the regression model for the propensity score weights) [15]. Our approach to simultaneously account for nonresponse bias and potential confounding factors, while a strength of the current study, does not preclude the possibility that unmeasured confounding factors contribute to our findings. Neither the Casas, Tiesler [14] or the Miles, Parast [15] study, however, examined potential sex-specific effects of pet keeping on the strengths and difficulties questionnaire or ADHD, making us unable to compare our sex-specific findings to previous studies.

We and others have shown that pets alter the microbiome of house dust [47–49]. We have also shown that prenatal exposure to pets alters the gut microbiome of neonates at approximately 1 month of age [17]. Initial colonizers of the gut play an essential role in establishment and maturation of the gut microbiome, which reaches adult stage at approximately 3–5 years of age [50]; differences in early colonizers could alter the trajectory of the gut microbiome and influence both current and future health. A small but growing body of literature suggests that the gut microbiome may influence the risk of ADHD [19, 20]. In a cross-sectional study of treatment naïve children in China, children with ADHD had lower levels of *Faecalibacterium*, *Dialister* and *Sutterella* than healthy controls, and lower levels of *Faecalibacterium* were associated with increased ADHD symptom severity [20]. Consistent with these findings, data from the WHEALS cohort showed that the keeping of indoor pets was associated with lower abundance of *Faecalibacterium* in neonatal stool at age ~ 1 month [17]. Mechanistically, the human gut microbiome is capable of producing a vast range of bioactive metabolites that could influence neurodevelopment and ADHD, potentially via upregulation of proinflammatory cytokines, altered mitochondrial function and blood-brain barrier or gut permeability, or through stimulating afferent endings of the vagus nerve [51–54]. Future studies evaluating potential mediating effects of the gut microbiome on the association of prenatal pet keeping and ADHD could further illuminate the pathway.

Prenatal pet keeping may also influence child neurodevelopment via mechanisms other than the gut microbiome. Pets could be a conduit for other environmental exposures, either through the application of pesticides to the pet itself (e.g., flea or tick medications) or by introduction of outdoor pesticides or toxicants into the home. For example, in the Childhood Autism Risks from Genetics and the Environment (CHARGE) study, a population-based study of children in California with ASD, developmental delays, and children from the general population, children with ASD had higher odds of having prenatal exposure to the flea and tick medication imidacloprid [16]. Pet dogs have been shown to act as a vehicle between outdoor pesticide application (diazinon) and introduction of these residues in the home [22]. A recent systematic review showed consistent evidence that prenatal exposure to organophosphate pesticides, which includes diazinon, is associated with adverse neurodevelopment across childhood; proposed mechanisms linking organophosphate pesticide exposure with negative impacts on neurodevelopment include impacting synaptic formation in utero or development of brain anomalies (e.g. damage to neurons or alterations in size of specific brain regions) [55]. Alternatively, Endenburg and van Lith [24], in a review on the influence of animals on child development, describe that it may not be the ownership of a pet, but rather the attachment to a pet that may influence emotional development. We do not have information on flea or tick medications used in the prenatal period, nor on the maternal or WHEALS child attachment to the pets in the home. Future studies that evaluate pet medications and attachment to and type of interaction with pets is needed.

Our study has a number of strengths and limitations. The prevalence of ADHD in WHEALS is comparable to the prevalence of ADHD reported in Michigan in 2011 (14.5% versus 12.8%) [56]. It is unlikely our results are due to reverse causality, as the keeping of pets prenatally would not be influenced by offspring behavior in childhood. WHEALS is a longitudinal birth cohort established in the prenatal period. Data on prenatal pet keeping was asked during the WHEALS prenatal interview, thus we do not have issues of recall bias, which has been an issue in other studies assessing prenatal risk factors for ADHD [32]. We do not have data on family history of ADHD. Given that ADHD is highly heritable [28], it is possible our parameter estimates for pet keeping with ADHD are biased by not accounting for family history or genetic risk. We did not have information on ADHD subtype; future analyses that obtain additional phenotypic information are needed. Our outcomes were caregiver-reported, which places us at risk for self-report bias. However, because most schools require a physician’s diagnosis (in writing) for a child to be considered potentially eligible for special education [57], it is likely in our sample that most caregiver reports of ADHD were based on medical diagnosis. Consistent with this, in our subsample of WHEALS children who had medical record abstraction, there was near perfect agreement between caregiver-reported and medical record documented ADHD.

## Conclusions

In conclusion, we found evidence that maternal exposure to dogs prenatally is positively associated with caregiver-reported ADHD in boys at ages 10–12 years. Future studies that replicate these findings are needed, as are studies that evaluate potential mechanisms linking these factors (e.g., the microbiome).

## Data Availability

The datasets analysed for this study are available from the corresponding author on reasonable request and with necessary permissions.

## Abbreviations

ADHD: Attention deficit hyperactivity disorder
ASD: Autism spectrum disorder
BMI: Body mass index
CI: Confidence interval
IPW: Inverse probability weights
NT: Neurotypical
OR: Odds Ratio
SPD: Sensory processing disorder
WHEALS: Wayne County Health, Environment, Allergy and Asthma Longitudinal Study
